# Effective single-drug approach is welcomed for deep-vein thrombosis treatment

**Published:** 2010-09-01

## Rivaroxaban protection is non-inferior and safe when compared to low-molecular weight heparin and warfarin

Results from the EINSTIEN-DVT trial were announced at the recent ESC 2010 congress, showing the therapeutic equivalence of rivaroxaban (15 mg twice daily for three weeks, followed by 20 mg once daily) to current standard therapy in treating and preventing further venous thrombotic events (VTE).

Prof Harry Buller, Academic Medical Centre, Amsterdam presented the results in the hot-line session and pointed out that this trial is the largest ever undertaken in deep-vein thrombosis (DVT), with 3 449 patients entered into the study from 32 countries.

‘EINSTEIN-DVT was designed as a non-inferiority trial and although open label, all events were adjudicated blindly. The primary-efficacy outcome was defined as symptomatic recurrent VTE: composite of recurrent DVT, non-fatal or fatal pulmonary embolism (PE)’, he said.

This trial has now shown that rivaroxaban taken orally is as effective in preventing recurrence of symptomatic VTE as well-managed patients receiving the current standard therapy of injectable low-molecular weight heparin (LMWH), enoxaprin or fondaparinux, and an oral vitamin K antagonist, chiefly warfarin.

While scientific publication of the full results will still follow, Prof Buller presented substantiating data for the conclusions. ‘The first symptomatic recurrent VTE occurred in 3% of patients receiving standard therapy, compared to 2.1% on rivaroxaban (hazard ratio of 0.68). This difference was significant and reached our preset standard for non-inferiority’, he said.

‘Importantly, there was no difference in the principal safety outcome, measuring a composite of major and non-major clinically relevant bleeding events (8.1% in both treatment arms). Rivaroxaban did however show a tendency to cause fewer major bleeds (14 in the rivaroxaban arm and 20 in the enoxaparin/warfarin arm)’, Prof Büller said. Net clinical benefit, a parameter of primary efficacy plus no major bleeding, favours rivaroxaban therapy.

Overall control of patients receiving enoxaprin and vitamin K antagonists was good, with 57% within the targeted INR range of 2 to 3, and this increased to 60% at three months after the trial. The trial was conducted over a 12-month period.

Discussing the results, Eve Knight of Anti-Coagulant Europe, the non-profit organisation that represents the interests of patients who have to take anti-coagulant therapy following a VTE, pointed out that patients have great difficulty with the cumbersome warfarin therapy

‘You are continually having to visit the clinic and have blood drawn for INR monitoring. You have to watch your food and drink intake, which greatly impacts on quality of life. VTEs affect young people who are still very active, the patients in the EINSTEIN trial were about 56 years of age, causing ongoing disruption to one’s working life and can affect career progress very badly’, she stressed.

Rivaroxaban is available in South Africa for the prevention of DVT following orthopaedic surgery and Bayer will be making representation to regulatory authorities across the world for the use of this drug in treating both primary and secondary DVT. ‘As this drug has the potential to change clinical practice, it may well receive priority attention’, Dr Berkowitz, Bayer Schering Pharma medical director noted.

